# Study protocol of a randomized phase III trial of comparing preoperative chemoradiation with preoperative chemotherapy in patients with locally advanced gastric cancer or esophagogastric junction adenocarcinoma: PREACT

**DOI:** 10.1186/s12885-019-5728-8

**Published:** 2019-06-20

**Authors:** Xiaowen Liu, Jiejie Jin, Hong Cai, Hua Huang, Guangfa Zhao, Ye Zhou, Jianghong Wu, Chunyan Du, Ziwen Long, Yantian Fang, Mingze Ma, Guichao Li, Menglong Zhou, Jiliang Yin, Xiaodong Zhu, Ji zhu, Weiqi Sheng, Dan Huang, Hui Zhu, Zhaozhen Zhang, Qi Lu, Li Xie, Zhen Zhang, Yanong Wang

**Affiliations:** 1 Department of Gastric Cancer Surgery, Fudan University Shanghai Cancer Center, Shanghai, 200032 China; 2 Department of Oncology, Shanghai Medical College, Fudan University, Shanghai, 200032 China; 3 Department of Radiotherapy, Fudan University Shanghai Cancer Center, 270 Dong An Road, Shanghai, 200032 China; 4 Department of Medical Oncology, Fudan University Shanghai Cancer Center, Shanghai, 200032 China; 5 Department of Pathology, Fudan University Shanghai Cancer Center, Shanghai, 200032 China; 6 Department of Radiology, Fudan University Shanghai Cancer Center, Shanghai, 200032 China; 7 Department of Endoscopy, Fudan University Shanghai Cancer Center, Shanghai, 200032 China; 8 Department of General Surgery, Huadong Hospital Affiliated to Fudan University, Shanghai, 200040 China; 9 Clinical Research Center, Shanghai Jiao Tong University School of Medicine, Shanghai, 200025 China

**Keywords:** Gastric cancer, Esophagogastric junction adenocarcinoma, Preoperative chemotherapy, Preoperative Chemoradiation, Gastrectomy, Adjuvant chemotherapy

## Abstract

**Background:**

The prognosis of patients with locally advanced gastric cancer or esophagogastric junction adenocarcinoma is still dismal. There are no standard treatment strategies for these patients. Multidisciplinary team (MDT) approach is a good choice for making a high-quality decision. Generally, MDT will recommend these patients to receive preoperative chemotherapy or preoperative chemoradiation based on all kinds of treatment guidelines. However, the preferred preoperative treatment is still not established. In order to solve this problem, we carry out this randomized phase III trial of comparing preoperative chemoradiation with preoperative chemotherapy in patients with locally advanced gastric cancer or esophagogastric junction adenocarcinoma.

**Methods:**

Eligible patients with locally advanced gastric cancer or esophagogastric junction adenocarcinoma are randomized to receive preoperative chemoradiation or preoperative chemotherapy, followed by surgery and postoperative chemotherapy. In the preoperative chemoradiation arm (Pre-CRT), patients receive two cycles of S-1 and oxaliplatin (SOX), chemoradiation, then followed by surgery and three more cycles of SOX chemotherapy. In the preoperative chemotherapy arm (Pre-CT), patients receive three cycles of SOX, following surgery three more cycles of SOX are given. The primary endpoint of this trial is to verify that preoperative chemoradiation could significantly improve the 3-year disease free survival (DFS) of patients with locally advanced gastric cancer or esophagogastric junction adenocarcinoma compared to preoperative chemotherapy.

**Discussion:**

The results from this trial will provide important information about whether preoperative chemoradiation could improve survival compared to preoperative chemotherapy among patients with locally advanced gastric cancer or esophagogastric junction adenocarcinoma.

**Trial registration:**

ClinicalTrials.gov Identifier: NCT03013010. First posted January 6, 2017.

## Background

Gastric cancer is still the sixth most common cancer around the world, and approximately 40% of new patients with gastric cancer are diagnosed in China [1]. Surgery is still the most effective treatment modality. However, most Chinese patients with gastric cancer lose the chance to undergo surgery due to being diagnosed as advanced stage. Among these patients, those with locally advanced stage still have potential opportunity to receive surgery after preoperative chemotherapy or chemoradiation, which is also recommended by the NCCN guideline [2]. Based on MAGIC and FNCLCC/FFCD trial, perioperative chemotherapy has been established as an alternative option for patients with resectable gastric cancer [3, 4]. Additionally, there are some preoperative chemoradiation reports in gastric cancer. A prospective, randomized trial showed that preoperative chemoradiation followed by surgery was superior to surgery in patients with resectable adenocarcinoma of the esophagus and gastric cardia [5]. Some phase II studies also showed that preoperative chemoradiation resulted in substantial pathologic response and R0 resection rate [6–8]. In our previous published research, 67% patients with locally advanced gastric cancer received curative gastrectomy after receiving chemoradiation [8]. Based on the publication of above-mentioned studies, clinicians have been faced with the dilemma of which preoperative therapy, chemotherapy or chemoradiation, should be applied to patients with locally advanced gastric cancer. A phase III study of comparing preoperative chemotherapy with chemoradiation was carried to solve this problem [9]. Unfortunately, this study was closed prematurely due to low accrual. Therefore, a randomized phase III trial comparing preoperative chemotherapy with chemoradiation is needed is deeply needed.

The aim of this study is to compare perioperative S-1 and oxaliplatin (SOX) chemotherapy with preoperative chemoradiation plus perioperative SOX chemotherapy in patients with locally advanced gastric cancer. Among the various chemotherapy regimes, S-1 and oxaliplatin (SOX) appears to be effective. Some studies have shown that SOX had a favorable tumor response rate with a relatively mild toxicity profile [10, 11].The study protocol of this trial, which has the acronym PREACT, is described in this article. This trial is an prospective, multicenter study be conducted in China.

## Methods/design

### Study setting

**Fig. 1:**
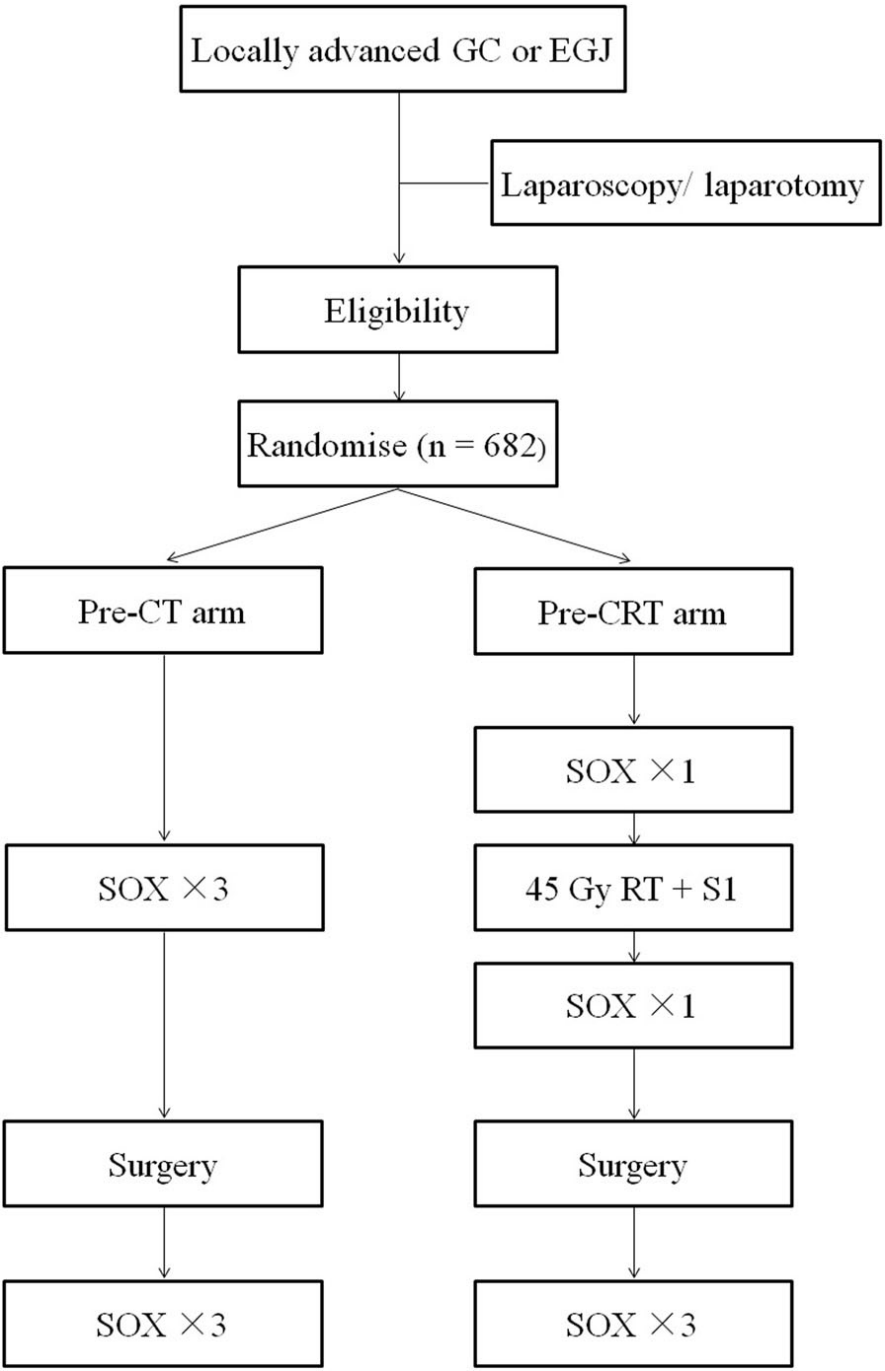
Flow chart

PREACT is a randomized phase III trial, which is carried out at multicenter in China. Eligible patients with locally advanced gastric cancer or esophagogastric junction adenocarcinoma are randomized to receive preoperative chemoradiation or preoperative chemotherapy, followed by surgery and postoperative chemotherapy (Fig. 1). Patients were randomized by the way of stratified permutated block randomization on Web-based system. Lauren classification is stratification factor. In Pre-CRT arm, patients receive two cycles of SOX plus chemoradiation, followed by surgery and three more cycles of SOX chemotherapy. In the Pre-CT arm, patients receive three cycles of SOX, following surgery three more cycles of SOX are given. The protocol has been approved by the Ethics Committee of Fudan University Shanghai Cancer Center, as well as other institutional ethics committees. All patients provided written informed consent before enrollment. Monitoring will be carried out in this trial.

### Primary endpoint

3-year disease free survival (DFS).

### Secondary endpoint

3-year overall survival (OS), curative gastrectomy rate, pathological response rate, treatment toxicity, and postoperative complications.

### Recruitment

Patients with locally advanced, histologically confirmed gastric cancer or esophagogastric junction adenocarcinoma are recruited. All the patients should take following examinations: chest radiography, CT scan, and endoscopic ultrasonography. Six hundred and eighty-two patients will be enrolled in China. If the patients meets all the inclusion criteria, staging laparoscopy or laparotomy will be performed to exclude peritoneal dissemination.

### Inclusion criteria

1. Age ≥ 18 years.
2. Eastern Cooperative Oncology Group performance status 0–1.
3. Life expectancy ≥6 months.
4. Histologically proven adenocarcinoma of the stomach or esophagogastric junction(excluding Siewert I).
5. Clinical stage is stage IIB (T3 N1 only), IIIA (T2 N3 not eligible), IIIB, and IIIC, i.e. T3 -T4a and node positive, or T4b and/ or node positive, according to American Joint Committee on Cancer (AJCC) 7th edition.
6. Considered to be potentially resectable verified by a multi-disciplinary team including a surgical investigator.
7. Patients have adequate organ function as follows:

- Haemoglobin ≥90 g/L, Absolute neutrophil count (ANC) ≥1.5 × 10^9^ /L, Platelet count≥100 × 10^9^ /L
- Serum bilirubin ≤1.5 × upper limit of normal, Aspartate aminotransferase (AST) and/or alanine transaminase (ALT) ≤2.5 × upper limit of normal
- Serum creatinine≤1.0 × upper limit of normal

8) Patients must provide the written informed consent.

### Exclusion criteria

1. Pregnant or lactating females or planning to become pregnant or lactating.
2. There is evidence of metastatic disease.
3. Prior chemotherapy or radiotherapy.
4. Patients have a history of cancer in the five years before randomization except for the squamous or basal cell carcinoma of the skin that has been effectively treated, and carcinoma in situ of the cervix that has been treated by operation.
5. Patients with central nervous system (CNS) disorder or peripheral nervous system disorder or psychiatric disease.
6. Patients with known history of uncontrolled or symptomatic angina, uncontrolled arrhythmias and hypertension, or congestive heart failure, or cardiac infarction within 6 months prior to study enrollment, or cardiac insufficiency.
7. Patients with severe infection.
8. Patients with severe gastrointestinal bleeding, gastrointestinal perforation, or unable to swallow.
9. Patients with known hypersensitivity reaction or metabolic disorder to S-1or oxaliplatin in this study.
10. Patients with linitis plastica.

### Treatments

#### Chemotherapy

**Fig. 2:**
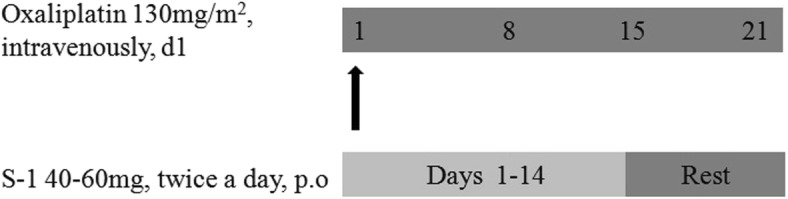
Chemotherapy schedule

SOX consists of S-1 and oxaliplatin. A course of chemotherapy will last 21 days. S-1 is administered orally with 40–60 mg, twice a day for 14 consecutive days, followed by a 7-day rest period (Fig. 2). The dose of S-1 was accorded to body-surface area (BSA): patients with a BSA of less than 1.25 m^2^ receive 80 mg daily; those with a BSA of 1.25 m^2^ or more but less than 1.5 m^2^ receive 100 mg daily; and those with a BSA of 1.5 m^2^ or more receive 120 mg daily. Oxaliplatin 130 mg/m^2^, intravenously, on day 1.

#### Chemoradiation

**Fig. 3:**
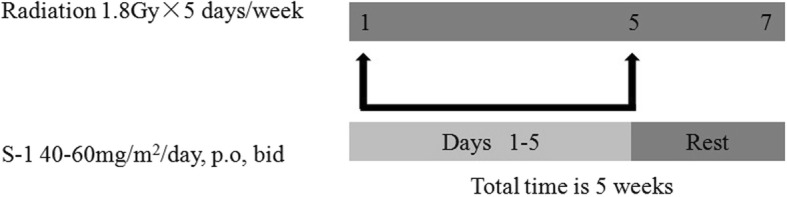
Chemoradiation schedule

Radiation is administered one week after completing the first cycle of chemotherapy. The treatment regimen consisted of 45 Gy of radiation delivered in 25 fractions ever five days per week for five weeks. Concurrent chemotherapy is S-1 40–60 mg/m^2^orally, oral tablet twice daily, days 1–5 of each week of radiotherapy (Fig. 3).Radiation treatment planning is done using computed tomography (CT) images. The radiation field includes the primary lesion and regional lymph node drainage.

#### Surgery

A standard D2 gastrectomy is recommended. The type of gastrectomy performed depends on the location and extent of the primary lesion. For middle third tumors, the gastric margin is recommended to be more than five cm, and a total gastrectomy is performed. For lower third tumors, a two cm duodenal margin is recommended and a subtotal or total gastrectomy is considered. For upper third tumors, a three cm esophageal margin is recommended and a total gastrectomy or esophagogastrectomy is performed. Billroth I or Roux-en-Y gastrojejunostomy is performed for distal gastrectomy patients, Roux-en-Y esophagojejunostomy is performed for patients receiving total gastrectomy.

#### Tumor response and toxicity criteria

In the preoperative chemoradiation arm, tumor response evaluations are taken after chemoradiation and second circle of preoperative SOX by using abdominal CT scan. In the preoperative chemotherapy arm, abdominal CT scan was performed to evaluate tumor response after third cycle of preoperative SOX. All these evaluations are done according to the Response Evaluation Criteria for Solid Tumors (RECIST) 1.1. Adverse events were assessed according to Common Terminology Criteria for Adverse Event (CTCAE) v4.0.

#### Follow up

Follow-up of all patients was carried out according to our protocol (every three months for at least two years, every six months for years 3–5, then every 12 months for life). Physical examination, tumor marker examination, and chest radiography were given every 3 months. Ultrasound and CT scan were given alternately every 6 months, and CT scan should be given at first follow-up. Endoscopic examination was given every 1 year [12].

### Sample size calculation

In this study, patients with cT3N + M0, cT4aN + M0 or cT4bN−/+M0 are recruited. According to AJCC 7th edition, these patients belong to stage IIB, IIIA, IIIB, and IIIC. We found that the 3-year disease free survival (DFS) of same stage patients after D2 gastrectomy and adjuvant chemotherapy is 41% in our database. Based on CLASSIC study, which showed that patient with II or III could get a 14% increase of 3-year DFS in adjuvant chemotherapy arm comparing to alone surgery arm [13], we speculate that 3-year DFS will be 27% for such stage patients after receiving alone D2 gastrectomy. Based on MAGIC study, which showed that patient could get a 13% increase of 3-year DFS in perioperative chemotherapy arm comparing to alone surgery arm [3], we speculate that 3-year DFS will be 40% for such stage patients after receiving perioperative chemotherapy. This study assumes a 10% increase in 3-year DFS favoring preoperative chemoradiation arm. Accrual time is four years followed by three years’ follow up. Dropouts are set 10%. It shows that a sample size of 682 will be needed with a 5% type I error (both sides) for a statistical power of 80%.

### Statistical analysis

DFS is calculated from the date of randomization to the date of detected disease recurrence. Following events are defined as recurrence: primary cancer recurrence, newly diagnosed gastric cancer, and death. OS is calculated from the date of randomization to the date of death or date of last follow-up. Survival is estimated by using the Kaplan-Meier method, and differences between survival curves are examined with log-rank test. Fisher’s exact is used to compare patients’ characteristics between preoperative chemotherapy arm and preoperative chemoradiation arm. Other secondary endpoints, including curative gastrectomy rate, pathological response rate, toxic effects, and postoperative complications, are compared between two arms using chi-square test. All statistical tests are two sides. The level of significance was *P* < 0.05.

## Discussion

This study is conducted to compare preoperative chemotherapy with preoperative chemoradiation in order to address which one is superior treatment in patients with locally advanced gastric cancer. Although this is not the first phase III trial of comparing preoperative chemotherapy with preoperative chemoradiation around the World, it has some advantages. As we all know, there are two obstacles needed to be addressed for preoperative therapy. First, most patients can not finish the scheduled treatment due to therapy toxicity. In this study, we use a combination of S-1 and oxaliplatin with concurrent radiation therapy as a preoperative treatment. SOX regimen has been proven to be effective and low-toxic, and has become a standard treatment in some Asian countries [14]. Therefore, we hypothesize that it may improve the patients’ tolerance to chemotherapy. Second, clinical staging is not precise. Despite the fact that imaging techniques have substantially improved the diagnostic accuracy of infiltration and lymph node involvement, peritoneal dissemination can not be detected precisely. Especially, it is at high risk of intra-abdominal metastases in patients with locally advanced gastric cancer [15]. However, no clinical trials in gastric cancer mentioned how to solve this problem on the condition of preoperative chemotherapy or chemoradiation [3–7, 9, 16]. In this study, all the patients will receive exploratory laparotomy or laparoscopy and peritoneal cytological examination in order to exclude peritoneal carcinomatosis. Meanwhile, for patients with obstruction of gastric pylorus, we will perform by-pass surgery to improve the nutrition status.

In all, the results of this study will provide the first prospective multi-center data of comparing preoperative chemoradiation with chemotherapy in China, which will contribute to establish treatment standards for clinical practice in patients with locally advanced gastric cancer or esophagogastric junction adenocarcinoma.

## Abbreviations

BSA: Body-surface area
CT: Computed tomography
DFS: Disease free survival
Pre-CRT: Preoperative chemoradiation
Pre-CT: Preoperative chemotherapy
SOX: S-1 and oxaliplatin
